# Artificial Differences in *Clostridium difficile* Infection Rates Associated with Disparity in Testing

**DOI:** 10.3201/eid2403.170961

**Published:** 2018-03

**Authors:** Mini Kamboj, Jennifer Brite, Anoshe Aslam, Jessica Kennington, N. Esther Babady, David Calfee, Yoko Furuya, Donald Chen, Michael Augenbraun, Belinda Ostrowsky, Gopi Patel, Monica Mircescu, Vivek Kak, Roman Tuma, Teresa A. Karre, Deborah A. Fry, Yola P. Duhaney, Amber Moyer, Denise Mitchell, Sherry Cantu, Candace Hsieh, Nancy Warren, Stacy Martin, Jill Willson, Jeanne Dickman, Julie Knight, Kim Delahanty, Annemarie Flood, Jennifer Harrington, Deborah Korenstein, Janet Eagan, Kent Sepkowitz

**Affiliations:** Memorial Sloan Kettering Cancer Center, New York, New York, USA (M. Kamboj, J. Brite, A. Aslam, J. Kennington, N.E. Babady, D. Korenstein, J. Eagan, K. Sepkowitz);; New York-Presbyterian/Weill Cornell Medical Center, New York (D. Calfee);; Columbia University Medical Center, New York (Y. Furuya);; Westchester Medical Center, Westchester, New York, USA (D. Chen);; University of Brooklyn, Brooklyn, New York, USA (M. Augenbraun);; Montefiore Medical Center, New York (B. Ostrowsky);; Mount Sinai Hospital, New York (G. Patel);; St. Joseph Medical Center, Phoenix, Arizona, USA (M. Mircescu);; Henry Ford Allegiance Health, Jackson, Mississippi, USA (V. Kak);; Easton Hospital, Easton, Pennsylvania, USA (R. Tuma);; Methodist Hospital, Omaha, Nebraska, USA (T.A. Karre);; Lehigh Valley Health Network, Allentown, Pennsylvania, USA (D.A. Fry);; South Miami Hospital, Miami, Florida, USA (Y.P. Duhaney);; Hartford Hospital, Hartford, Connecticut, USA (A. Moyer, D. Mitchell);; Backus Hospital, Norwich, Connecticut, USA (A. Moyer, D. Mitchell);; Windham Hospital, Willimantic, Connecticut, USA (A. Moyer, D. Mitchell);; Mid State Medical Center, Meriden, Connecticut, USA (A. Moyer, D. Mitchell);; Hospital of Central Connecticut, New Britain, Connecticut, USA (A. Moyer, D. Mitchell);; MD Anderson Cancer Center, Houston, Texas, USA (S. Cantu);; Dana Farber Cancer Institute, Boston, Massachusetts, USA (C. Hsieh);; Fox Chase Cancer Center, Philadelphia, Pennsylvania, USA (N. Warren);; Moffitt Cancer Center, Tampa, Florida, USA (S. Martin);; University of Southern California Norris Comprehensive Cancer Center, Los Angeles, California, USA (J. Willson);; James Cancer Center, Columbus, Ohio, USA (J. Dickman);; Seattle Cancer Care Alliance, Seattle, Washington, USA (J. Knight);; University of California San Diego Hospital, San Diego, California, USA (K. Delahanty);; City of Hope, Duarte, California, USA (A. Flood);; Roswell Park, Buffalo, New York, USA (J. Harrington)

**Keywords:** Clostridium difficile, healthcare-associated infection, nucleic acid amplification tests, testing rate, National Healthcare Safety Network, bacteria, United States

## Abstract

In 2015, *Clostridium difficile* testing rates among 30 US community, multispecialty, and cancer hospitals were 14.0, 16.3, and 33.9/1,000 patient-days, respectively. Pooled hospital onset rates were 0.56, 0.84, and 1.57/1,000 patient-days, respectively. Higher testing rates may artificially inflate reported rates of *C. difficile* infection. *C. difficile* surveillance should consider testing frequency.

Persons testing positive for *Clostridium difficile* by molecular methods might not always have *C. difficile* disease. Up to 10% of hospitalized patients carry toxigenic *C. difficile*; carriage rates of 35%–50% have been described in certain high-risk settings (*1*–*4*). In contrast, the risk for *C. difficile* infection (CDI) among hospitalized patients is 1%–2% (*5*).

Nucleic acid amplification testing (NAAT) has revolutionized diagnostic microbiology. Rapid, highly sensitive results can be returned to clinicians within hours, helping them make timely management decisions. The exquisite sensitivity of the test, however, has created an unexpected problem for *C. difficile* diagnosis: the test cannot distinguish patients with active disease from those who are asymptomatic carriers. Several clinical studies have shown that persons who test positive by NAAT without concomitant detection of toxin on conventional assays have milder disease than toxin-positive persons (*6*–*8*). The conclusion from these studies is straightforward: NAATs are highly sensitive but have an unacceptably low positive predictive value. Unnecessary clinical testing will generate many false cases; several downstream sequelae are potentially detrimental for patients and add to superfluous healthcare-associated costs.

## The Study

We hypothesized that, in the era of NAAT-based testing for CDI, unevenness in testing rates introduces variability in measurement of CDI among different healthcare settings. We designed our survey to capture information about hospital characteristics and various aspects of *C. difficile* testing from a convenience sample of 38 medical centers, including 17 members of the Comprehensive Cancer Center Infection Control Network, 13 community hospitals, and 9 university-affiliated multispecialty centers. The survey consisted of 13 questions for the year 2015: total number of beds, admissions, oncology beds, and transplants; *C. difficile* testing practices (inpatient testing volume, diagnostic method, and rejection policies); and rate of hospital-onset CDI (HO-CDI). We compared rates using ordinary 1-way analysis of variance or Kruskal–Wallis and determined the Pearson coefficient to measure the strength and direction of linear relationships between testing and *C. difficile* infection rates. We considered p<0.05 as significant. Each hospital provided information after responding to the Institutional Review Board and Health Insurance Portability and Accountability Act considerations at its institution.

Of the 31 (82%) hospitals responding to the survey, 30 provided complete data (Table). Overall, NAAT use was 87% (82% of community hospitals, 100% of multispecialty centers, and 80% of cancer centers). Among centers with 2-step testing, the commonly used initial test was glutamate dehydrogenase with or without toxin enzyme immunoassay, followed by NAAT for indeterminate samples. The overall fecal positivity rate among centers that used a 1-step NAAT was 16% for community hospitals, 15% for multispecialty centers, and 12% for cancer centers. Among NAAT users, the positivity rate for 1-step versus 2-step tests did not differ (13.9% vs. 14.3%, respectively). Formed fecal samples were rejected at 28 of 30 hospitals. Fifteen centers implemented a formal policy to avoid testing replicate samples; 2 rejected samples from patients receiving laxatives.

**Table:** Hospital characteristics of study centers and methods used for the diagnosis of *Clostridium difficile* infection, 2015*

Characteristic	Hospital type, N = 30		
	Community hospital, n = 11	Large multispecialty academic center, n = 9	Tertiary-care cancer center, n = 10
			
Average no. beds (range)	294 (156–472)	869 (563–1,525)	221 (20–660)
No. annual admissions (range)	12,297 (2,897–22,000)	38,711 (14,589–86,658)	9308 (459–28,400)
No. annual patient-days (range)	56,322 (12,249–88,241)	241,034 (155,140–469,664)	65,263 (5,832–202,483)
Average length of stay, d	4.76	7.2	7.95
Transplantation, no.			
Hematopoietic stem cell	0	7	9
Solid organ	0	8	0
Diagnostic test, no.			
NAAT, 1-step	7	6	5
NAAT, 2-step	2	3	3
No. rejections of formed fecal samples	10	9	8
Pooled HO-CDI rate/1,000 patient-days	0.56	0.87	1.57

*HO-CDI, hospital-onset *C. difficile* infection; NAAT, nucleic acid amplification testing.

**Figure 1:**
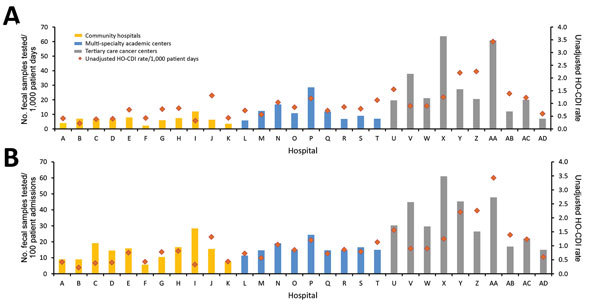
Hospital-specific rates of testing for *Clostridium difficile* standardized by patient-days of admission (A) and number of admissions (B), with HO-CDI rates (cases/1,000 patient-days), 30 US hospitals, 2015. HO-CDI, hospital-onset *C. difficile* infection.

We determined the testing rate for each study hospital (Figure 1). The mean number of tests per 100 admissions for 2015 was 6.4 for community hospitals, 12.2 for multispecialty centers, and 29.1 for cancer centers (p = 0.0003). The mean number of tests per 1,000 patient-days was 14.0, 16.3, and 33.9, respectively (p = 0.0016). A separate analysis on the subset of centers using PCR-based testing yielded similar results: 6.1, 12.6, and 28.0 tests/100 admissions (p = 0.0058) and 14.1, 16.3, and 33.4 tests/1,000 patient-days, respectively (p = 0.015).

**Figure 2:**
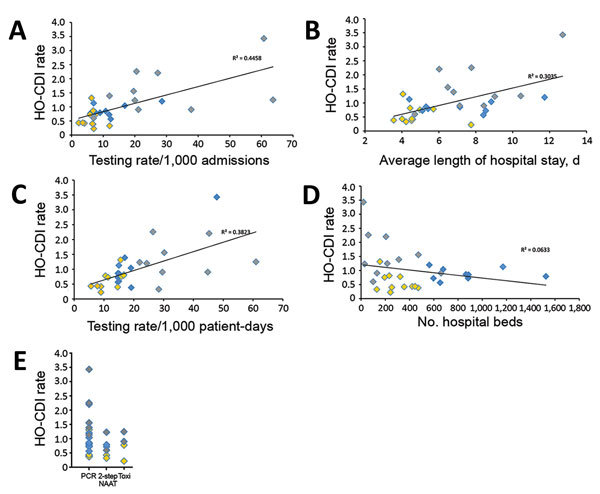
Correlation between HO-CDI rates (per 1,000 patient-days) and standardized testing volume (A,B), average length of hospital stay (C), number of hospital beds (D), and diagnostic test used (E), 30 US hospitals, 2015. Yellow indicates community hospitals; blue, multispecialty academic centers; gray, tertiary care cancer centers. p values and Pearson coefficient (*r*) values are as follows: A) p<0.001, *r* = 0.29. B) p = 0.0014, *r* = 0.57. C) p = 0.0003, *r* = 0.68. D) p = 0.1276, *r* = –0.29. EIA, enzyme immunoassay; HO-CDI, hospital-onset *C. difficile* infection; NAAT, nucleic acid amplification testing.

The mean rate of hospital-onset *C. difficile* infection for the community hospitals, multispecialty centers, and cancer centers was 0.57, 0.88, and 1.57 cases per 1,000 patient-days (p = 0.0007). The correlation between testing rates, number of hospital beds, and average length of stay (Figure 2, panels A–D) illustrated a positive linear relationship between testing rates and length of stay. HO-CDI rates were highest for cancer centers that use NAAT (Figure 2, panel E).

## Conclusions

We demonstrated that hospitals that test for *C. difficile* more frequently, such as cancer hospitals, have significantly higher CDI rates. This finding has 2 logical explanations. First, patients with cancer and patients in highly specialized hospitals have higher rates of HO-CDI because of the complexity of their underlying conditions and treatment. Second, and of particular importance in the era of reported healthcare-associated infections, overtesting can overdiagnose carriers of *C. difficile* as *C. difficile* cases, when in fact these patients have nondisease conditions of lesser clinical and epidemiologic significance.

Our study findings suggest that much of the excess *C. difficile* diagnosis in tertiary and cancer centers might be attributable to overtesting. We base our conclusion on the significant association found between testing rates and level of specialized care, particularly among cancer centers, as well as a positive correlation between testing and rates of HO-CDI. Among cancer centers, the likely explanation for excessive testing despite good diagnostic stewardship is the higher prevalence of diarrhea caused by effects of chemotherapy, newer immunotherapeutic modalities, and transplant-related gastrointestinal complications.

During the early 2000s, a hypervirulent NAP1 (North American pulsed-field gel electrophoresis type 1) *C. difficile* strain emerged in North America and Europe (*9*,*10*). With the premise that early and reliable detection of CDI will enhance control, the first Food and Drug Administration–approved NAATs for *C. difficile* became available in 2008 (*5*,*11*). NAAT use increased sharply during 2011−2013. As of 2016, ten different molecular tests for *C. difficile* have been Food and Drug Administration approved. More than half of all acute care settings that report CDI rates to the National Healthcare Safety Network use NAAT-based testing (*12*). Reports of oversensitivity of NAAT followed the increase in use, leading to a 43%–100% increase in reported incidence rates of CDI (*7*,*11*,*13*).

From a reporting perspective, risk adjustments have been made during interfacility comparisons of rates of HO-CDI to account for differences in diagnostic testing methods (*11*). Despite the apparent association between testing rates and likelihood of false-positive results with NAATs, testing frequency for *C. difficile* has been overlooked as a reporting metric by the National Healthcare Safety Network.

Based on the most recent estimates from 2014, rates of HO-CDI have declined by 8% in the United States since 2011 (*14*). Whether some component of this is due to less testing than in previous years remains unknown. Hospitals that use NAAT that have performed better over time may have truly reduced infections or simply adjusted to the new test. Current surveillance methods do not consider the effect of testing volume on reported *C. difficile* rates, a common cause of artificial changes.

Our report has several limitations. First, we used a convenience sample of 30 hospitals in which cancer centers were overrepresented. Second, we did not include clinical characteristics of patients tested for *C. difficile* because our survey focused on comparing testing rates in several representative hospitals across the healthcare continuum.

In conclusion, CDI infection surveillance programs must recognize that testing methods and testing frequency need to be considered independently when comparing infection rates. In addition, frequency of diarrhea in hospitalized patients is an important determinant that might vary by patient–case mix and affect testing and rates of HO-CDI. Testing frequency is not only important for local quality improvement but also should be made an essential component of *C. difficile* reporting to standardize disease measurement, monitor effectiveness of prevention strategies, and establish meaningful trends.

## References

[1] Curry SR, Muto CA, Schlackman JL, Pasculle AW, Shutt KA, Marsh JW, et al. Use of multilocus variable number of tandem repeats analysis genotyping to determine the role of asymptomatic carriers in *Clostridium difficile* transmission. Clin Infect Dis. 2013;57:1094–102. 10.1093/cid/cit475 23881150 PMC3783061

[2] Kamboj M, Sheahan A, Sun J, Taur Y, Robilotti E, Babady E, et al. Transmission of *Clostridium difficile* during hospitalization for allogeneic stem cell transplant. Infect Control Hosp Epidemiol. 2016;37:8–15. 10.1017/ice.2015.237 26486102 PMC4896642

[3] Kinnebrew MA, Lee YJ, Jenq RR, Lipuma L, Littmann ER, Gobourne A, et al. Early *Clostridium difficile* infection during allogeneic hematopoietic stem cell transplantation. PLoS One. 2014;9:e90158. 10.1371/journal.pone.0090158 24662889 PMC3963842

[4] Riggs MM, Sethi AK, Zabarsky TF, Eckstein EC, Jump RL, Donskey CJ. Asymptomatic carriers are a potential source for transmission of epidemic and nonepidemic *Clostridium difficile* strains among long-term care facility residents. Clin Infect Dis. 2007;45:992–8. 10.1086/521854 17879913

[5] Lessa FC, Winston LG, McDonald LC; Emerging Infections Program C. difficile Surveillance Team. Burden of Clostridium difficile infection in the United States. N Engl J Med. 2015;372:2369–70. 10.1056/NEJMoa1408913 26061850 PMC10880113

[6] Beaulieu C, Dionne LL, Julien AS, Longtin Y. Clinical characteristics and outcome of patients with *Clostridium difficile* infection diagnosed by PCR versus a three-step algorithm. Clin Microbiol Infect. 2014;20:1067–73. 10.1111/1469-0691.12676 24813402

[7] Longtin Y, Trottier S, Brochu G, Paquet-Bolduc B, Garenc C, Loungnarath V, et al. Impact of the type of diagnostic assay on *Clostridium difficile* infection and complication rates in a mandatory reporting program. Clin Infect Dis. 2013;56:67–73. 10.1093/cid/cis840 23011147

[8] Polage CR, Solnick JV, Cohen SH. Toxin immunoassays and *Clostridium difficile* infection—reply. JAMA Intern Med. 2016;176:414–5. 10.1001/jamainternmed.2015.8539 26954051

[9] Loo VG, Bourgault AM, Poirier L, Lamothe F, Michaud S, Turgeon N, et al. Host and pathogen factors for *Clostridium difficile* infection and colonization. N Engl J Med. 2011;365:1693–703. 10.1056/NEJMoa1012413 22047560

[10] McDonald LC, Killgore GE, Thompson A, Owens RC Jr, Kazakova SV, Sambol SP, et al. An epidemic, toxin gene-variant strain of *Clostridium difficile.* N Engl J Med. 2005;353:2433–41. 10.1056/NEJMoa051590 16322603

[11] Gould CV, Edwards JR, Cohen J, Bamberg WM, Clark LA, Farley MM, et al.; Clostridium difficile Infection Surveillance Investigators, Centers for Disease Control and Prevention. Effect of nucleic acid amplification testing on population-based incidence rates of Clostridium difficile infection. Clin Infect Dis. 2013;57:1304–7. 10.1093/cid/cit492 23899677 PMC9580544

[12] Food and Drug administration. Nucleic acid based tests [cited 2017 Feb 13]. http://www.fda.gov/MedicalDevices/ProductsandMedicalProcedures/InVitroDiagnostics/ucm330711.htm

[13] Fong KS, Fatica C, Hall G, Procop G, Schindler S, Gordon SM, et al. Impact of PCR testing for *Clostridium difficile* on incident rates and potential on public reporting: is the playing field level? Infect Control Hosp Epidemiol. 2011;32:932–3. 10.1086/661789 21828981

[14] Centers for Disease Control and Prevention. Healthcare-associated infections. HAI progress report FAQ [cited 2017 Feb 13]. https://www.cdc.gov/hai/surveillance/progress-report/faq.html

